# Controlling Chaotic Resonance using External Feedback Signals in Neural Systems

**DOI:** 10.1038/s41598-019-41535-0

**Published:** 2019-03-21

**Authors:** Sou Nobukawa, Natsusaku Shibata

**Affiliations:** Department of Computer Science, Chiba Institute of Technology, 2-17-1 Tsudanuma, Narashino, Chiba 275-0016 Japan

## Abstract

Stochastic resonance is a phenomenon in which the signal response of a non-linear system is enhanced by appropriate external noise. Likewise, a similar phenomenon can be caused by deterministic chaos; this is called chaotic resonance. Devices that employ stochastic resonance have been proposed for the purpose of enhancing tactile sensitivity. However, no applications of chaotic resonance have been reported so far, even though chaotic resonance exhibits a higher sensitivity than stochastic resonance. This contrast in applications could be attributed to the fact that chaotic resonance is induced by adjusting internal parameters. In many cases, especially in biological systems, these parameters are difficult to adjust. In this study, by applying our proposed reduced region of orbit method to a neural system consisting of excitatory and inhibitory neurons, we induce chaotic resonance with signal frequency dependency against weak input signals. Furthermore, the external noise exhibits effects for both diminishing and enhancing signal responses in chaotic resonance. The outcome of this study might facilitate the development of devices utilising the mechanism of chaotic resonance.

## Introduction

Over the past few decades, resonance and synchronisation phenomena in various kinds of non-linear systems, such as chemical, biological, and electrical circuit systems, have been widely investigated^1–3^. It is known that these resonance phenomena and the existence of fluctuations may enhance system functionalities^4–11^. In particular, fluctuations in neural activity, such as noise and chaos, are widely observed at several hierarchical levels–from the intra-neuronal level to brain activity levels–and could enhance neural information processing^12–14^. One of the mechanisms for enhancing brain functionality with fluctuations, stochastic resonance, is a phenomenon in which the signal response of a non-linear system is enhanced by external noise with appropriate strength^15–18^. Consider an example of stochastic resonance in sensory neural systems: crayfish and paddlefish detect slight movements of predators and prey using background noise^16–18^. Stochastic resonance also arises in the visual processing area of the human brain in the experimental condition, wherein brain waves are entrained by light stimuli^14,19^. Moreover, the strength of fluctuations in brain activity correlates with cognitive functions, that is, the optimised strength of neural fluctuation; the mechanism of stochastic resonance may play a vital role in neural processing in the brain^20–23^.

In addition to stochastic noise, deterministic chaos also causes a similar phenomenon, called chaotic resonance^2,24–29^. Here, two kinds of conditions for chaotic resonance have been considered. The first is the case wherein the external deterministic chaotic signal is applied to the system instead of the external stochastic noise^24,25,29^. Specifically, the external chaotic signal, which is produced by chaotic systems, such as tent maps and logistic maps, is applied to a bi-stable dynamic system with a weak input signal^24,25,29^. In the second case, intrinsic chaotic dynamics are utilised instead of external additive chaotic signals (hereinafter, we address this type of chaotic resonance)^2,25–28^. This type of chaotic resonance is mainly observed in chaotic systems with chaos–chaos intermittency, wherein the orbit hops among separated regions^2^. In these systems, the timing of chaos–chaos intermittency is synchronised with weak external input signals by adjusting internal system parameters. At first, this phenomenon was evaluated in simple models and electrical circuits, such as one-dimensional cubic maps, Lorenz models, and Chua’s circuit^2,26–28,30^. Since these studies, the exploration of chaotic resonance in neural system has progressed^31–34^.

With regard to the application of these resonances, Kurita *et al*. developed a wearable device for enhancing tactile sensitivity by applying white noise vibration to fingertips, utilising the mechanism of stochastic resonance^6,8^. Enders *et al*. proposed a method to improve haptic sensations for paralysed patients with additive vibrotactile noise^35^. Seo *et al*. applied this type of method to the rehabilitation of stroke survivors^36^. However, no applications have yet been reported for chaotic resonance despite the fact that it exhibits a higher sensitivity than stochastic resonance^32^. This discrepancy is because stochastic noise can be easily adjusted and controlled to the appropriate strength for inducing stochastic resonance, whereas to induce chaotic resonance, chaos must be adjusted to the appropriate chaotic state by adjusting an internal parameter of the system. In the case of biological systems in particular, this adjustment is difficult.

To overcome this difficulty, we have previously proposed a chaos control method called the reduced region of orbit (RRO) method, which induces chaotic resonance using external feedback signals^37^. Specifically, the RRO method realises attractor merging by reducing the range of the chaotic orbit. Compared with conventional chaos control methods, in which the chaotic state is eliminated by stabilising equilibrium and transiting to a periodic state through external perturbation (as in the Ott–Grebogi–Yorke method^38^, namely the delayed feedback method^39,40^ and *H*_∞_ control^41^), this method does not remove the chaotic state. Rather, it adjusts the appropriate frequency of chaos–chaos intermittency–for chaotic resonance to arise^37^. However, the RRO method has only been adopted for a cubic map system as a simple discrete chaotic system^37,42^. To realise the application of the RRO method for inducing chaotic resonances in actual biological systems, its adoption to models for neural systems must be considered. Their dynamics of models for neural system have been widely investigated for continuous spiking neuron models, spiking neuron models with discontinuous resetting processes (called hybrid spiking neuron models), and discrete neural system models^43–49^. Among these neural models, it is known that a discrete neural system consisting of excitatory and inhibitory neurons, as proposed by Sinha, has a structure similar to that of the cubic map, and chaotic resonance arises by adjusting the internal system parameters^31^.

In this work, the RRO method is applied to the discrete neural system proposed by Sinha^31^. The chaotic resonance induced by the RRO method is evaluated in terms of the feedback strength of the method, internal neural parameters, input signal parameters, and robustness of the presence of stochastic noise.

## Material and Methods

### Neuron model

**Figure 1:**
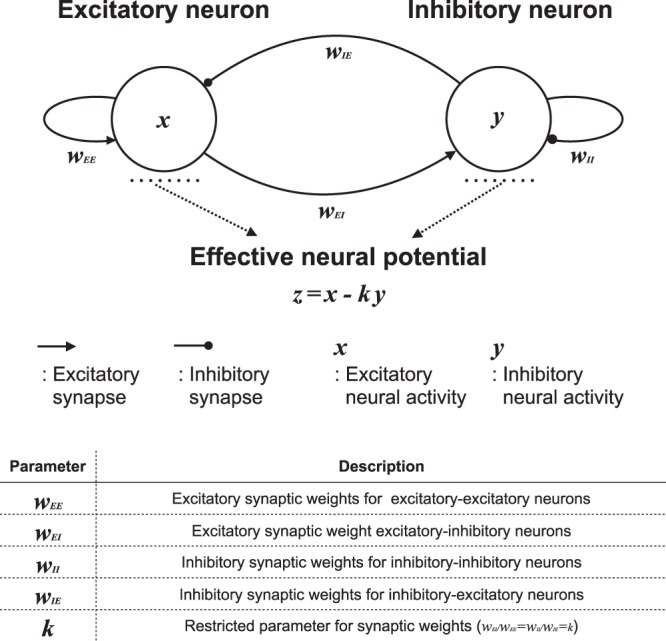
Overview of neural system consisting of excitatory and inhibitory neurons proposed by Sinha^31^ and description of its variables and parameters.

In this study, we use the discrete neural system consisting of excitatory and inhibitory neurons proposed by Sinha^31^. As shown in Fig. 1, this neural system consists of excitatory and inhibitory neurons coupled by excitatory and inhibitory synaptic connections: excitatory synaptic weights between excitatory neurons (*w*_*EE*_) and from excitatory neurons to inhibitory neurons (*w*_*EI*_), and inhibitory synaptic weights between inhibitory neurons (*w*_*II*_) and from inhibitory neurons to excitatory neurons (*w*_*IE*_). The state dynamics of the excitatory and inhibitory neurons, *x*(*t*) and *y*(*t*), respectively, are given by

1 $$x(t+\mathrm{1)}={F}_{a}({w}_{EE}x(t)-{w}_{EI}y(t)),$$

2 $$y(t+\mathrm{1)}={F}_{b}({w}_{IE}x(t)-{w}_{II}y(t)),$$

where functions *F*_*a*,*b*_ follow *F*_*a*_(*X*) = −1 (*X* < −1/*a*), *F*_*a*_(*X*) = *aX* (−1/*a* ≤ *X* ≤ 1/*a*), *F*_*a*_(*X*) = 1 (*X* > 1/*a*), *F*_*b*_(*Y*) = −1 (*Y* < −1/*b*), *F*_*b*_(*Y*) = *bY* (−1/*b* ≤ *Y* ≤ 1/*b*), and *F*_*b*_(*Y*) = 1 (*Y* > 1/*b*). The dynamics of the effective neural potential *z* = *x* − *ky* under the restriction of *w*_*EI*_/*w*_*EE*_ = *w*_*II*_/*w*_*IE*_ = *k* is given by

3 $$z(t+\mathrm{1)}=F(z(t))={F}_{a}(z(t))-k{F}_{b}(z(t\mathrm{))}.$$

To control the chaos–chaos intermittency for *z*(*t*), the RRO feedback term *u*(*z*)^37^ is applied as follows:

4 $$z(t+\mathrm{1)}=F(z(t))+Ku(z(t)),$$

5 $$u(z)=-\,(z-{z}_{d})\exp (\,-\,{(z-{z}_{d})}^{2}/(2{\sigma }^{2})),$$

where *K*, *z*_*d*_, and *σ* indicate the feedback strength, the point dividing the chaotic attractor, and the parameter related to the range of the feedback control effect, respectively. In this study, we use the following parameter set for the neural system:*a* = 6.02, 6.03, 6.04; *b* = 3.42; *and k* = 1.3811^31^. In this parameter set, the orbit of *z*(*t*) exhibits a symmetric chaotic attractor about *z*(*t*) = 0, and hops between the positive and negative *z*(*t*) regions (chaos–chaos intermittency). According to our previous work^37^, *z*_*d*_ and *σ* are set to the divided points of each chaotic region *z*_*d*_ = 0, and the distance between the divided points and the local maximum/minimum points *σ* = 1/*a*.

**Figure 2:**
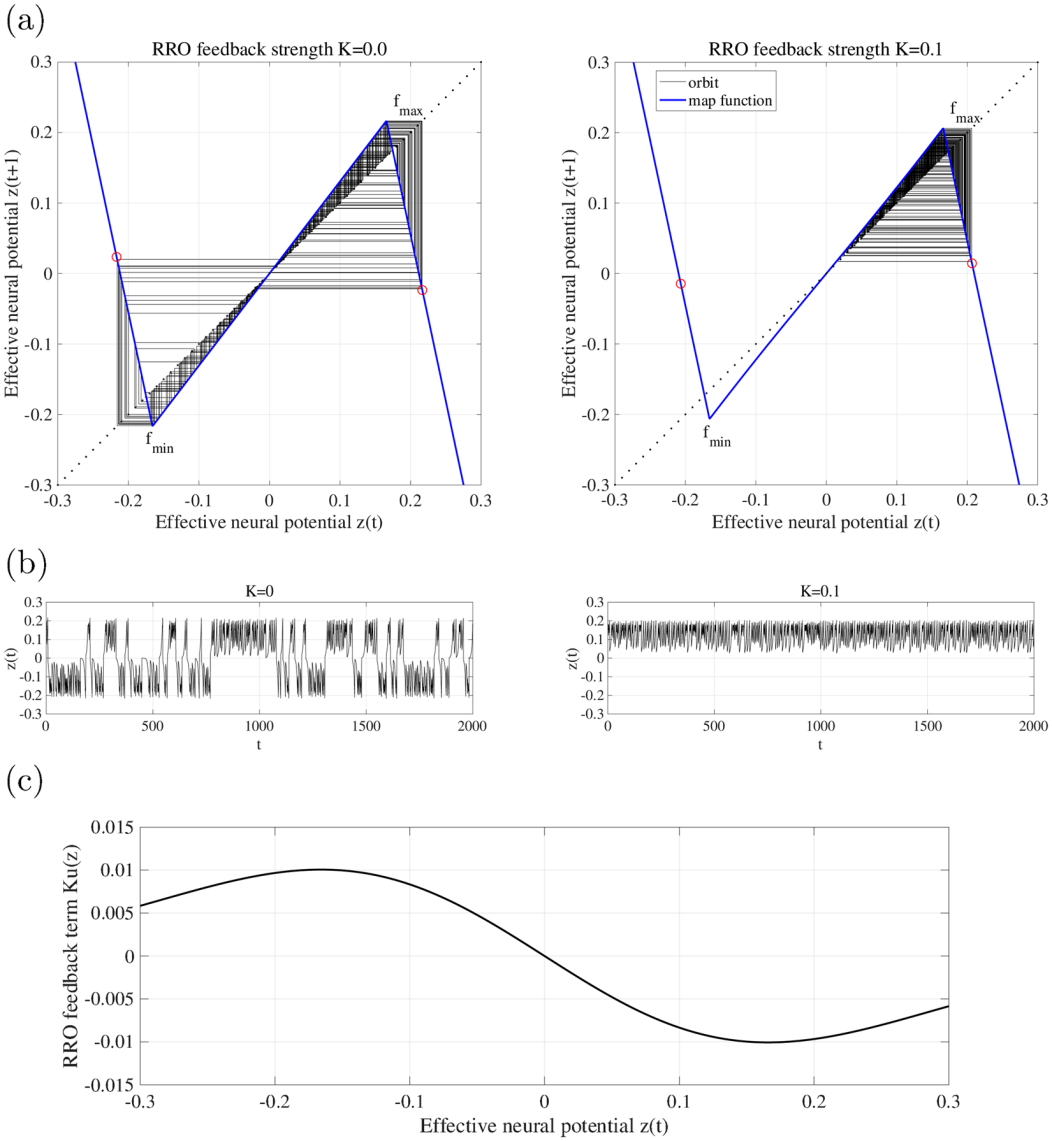
Effect of the reduced region of orbit (RRO) method for separating the merged attractor. (**a**) Map function of effective neural potential *z*(*t*) in the coupled neural system (blue solid line) and its orbit (black solid line) for *a* = 6.02 without RRO feedback (*K* = 0 (left)) and with RRO feedback (*K* = 0.1 (right)). The red circle indicates *F*(*f*_max,min_) + *K(u*(*f*_max,min_)) (*f*_max,min_, that is, local maxima and minima for the map function). (**b**) Time series of $z(t)$ without RRO feedback (*K* = 0 (left)) and with RRO feedback (*K* = 0.1 (right)). (**c**) Profile of the RRO feedback term given by eq. (5). Applying the RRO feedback control, the absolute values of *f*_max,min_ are reduced. Then, the attractor merging condition is broken: (*F*(*f*_max_) ≈ 0.17 > 0 and *F*(*f*_min_) ≈ 0.17 < 0). Consequently, the orbit is confined to one of the regions (either the positive or the negative region).

Let us demonstrate the mechanism of the RRO method for separating the merged attractor. Figure 2 shows the map function of *F* and the orbit of *z*(*t*) for *a* = 6.02 both without RRO feedback control (*K* = 0) and with it (*K* = 0.1). In the case without RRO feedback control, the orbits of *z*(*t*) go back and forth between the negative and positive regions because the condition for attractor merging, given by *F*(*f*_max_) < 0 and *F*(*f*_min_) > 0, where *f*_max,min_ are the local maxima and minima of *F*(*z*)^37,42^, is satisfied in this parameter set. RRO feedback control given by eq. (5) has local maxima and minima at the value of *z* approximately, where the map function *F* exhibits local minima and maxima, respectively (see Fig. 2(c)). Therefore, applying the RRO feedback control, the absolute values of *f*_max,min_ are reduced, as shown in the case with feedback control (*K* = 0.1) (right panel of Fig. 2(a))). Then, the attractor merging condition is broken: (*F*(*f*_*max*_) ≈ 0.17 > 0 and *F*(*f*_*min*_) ≈ −0.17 < 0). Consequently, the orbit is confined to one of the regions (either positive or negative), depending on the initial value of *z*(0).

To evaluate chaotic resonance, the sinusoidal signal *S*(*t*) = *A*sin 2*πωt* is applied:

6 $${z}(t+\mathrm{1)}=F(z(t))+Ku(z(t))+S(t\mathrm{)}.$$

Further, to evaluate the influence of external noise on the signal response for chaotic resonance, Gaussian white noise *ξ*(*t*) (mean value: 0, standard deviation: 1.0) is applied to eq. (6) as follows:

7 $$z(t+\mathrm{1)}=F(z(t))+Ku(z(t))+S(t)+D\xi (t),$$

where *D* indicates the noise strength.

### Evaluation index

The signal response was evaluated using the correlation coefficient (with time delay *τ*) between the binarised *z*(*t*) time series *Z*(*t*) (*Z*(*t*) = 1, if *z*(*t*) ≥ 0; *Z*(*t*) = −1, if *z*(*t*) < 0) and the time series of the input signal *S*(*t*), as follows:

8 $$C(\tau )=\frac{{C}_{SZ}(\tau )}{\sqrt{{C}_{SZ}{C}_{ZZ}}},$$

9 $${C}_{SZ}(\tau )=\langle (S(t+\tau )-\langle S\rangle )(Z(t)-\langle Z\rangle )\rangle ,$$

10 $${C}_{SS}=\langle {(S(t)-\langle S\rangle )}^{2}\rangle ,$$

11 $${C}_{ZZ}=\langle {(Z(t)-\langle Z\rangle )}^{2}\rangle ,$$

where 〈·〉 denotes the average in *t*.

To evaluate the chaos for the dynamics of *Z*(*t*), we use the Lyapunov exponent^50^:

12 $$\lambda =\frac{1}{\tau M}\sum _{k=1}^{M}{\rm{l}}{\rm{n}}(\frac{{d}^{k}({t}_{l}=\tau )}{{d}^{k}({t}_{l}=\mathrm{0)}}).$$

here, *d*^*k*^(*t*_*l*_ = 0) = *d*_0_ (*k* = 1, 2, …, *M*) denotes *M* perturbed initial conditions to *Z*(*t*) applied at *t* = *t*_0_ + (*k* − 1)*τ*. Their time evolution for *t*_*l*_ ∈ [0 : τ] is ${d}^{k}({t}_{l}=\tau )=(Z(t)-Z^{\prime} (t{))|}_{t={t}_{0}+k\tau }$. Further, *Z*′(*t*) denotes an orbit-applied perturbation.

To confirm the effect suppressing the frequency of chaos–chaos intermittency by RRO feedback, we used the corresponding probability, as follows:

13 $${P}_{t}={f}_{cc}/T,$$

where *f*_*cc*_ and *T* denote the frequency of chaos–chaos intermittency and the number of iterations, respectively.

## Results

### Controlling attractor merging

**Figure 3:**
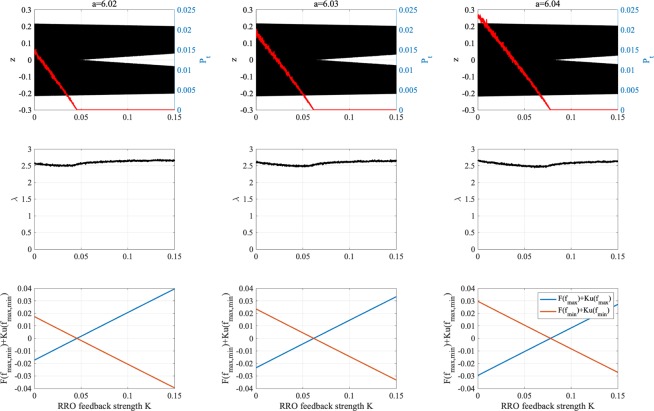
Dependence of system behavior on feedback strength *K*. Bifurcation diagram of effective neural potential *z*(*t*) (black dot) and probability of chaos–chaos intermittency *P*_*t*_ (red line) as a function of *K* with the RRO method (top). The negative and positive initial values *z*(0) are used. Dependence of the Lyapunov exponent *λ* (middle) and *F*(*f*_max,min_) + *K(u*(*f*_max,min_)) (bottom) on *K*. The chaotic attractor is divided owing to the broken attractor merging condition: *F*(*f*_max_) + *K(u*(*f*_max_)) > 0, *F*(*f*_min_) + *K(u*(*f*_min_)) < 0.

To observe the dependence of the system behaviour on the feedback strength *K*, Fig. 3 shows the bifurcation diagram of *z*(*t*) and *P*_*t*_ as a function of *K* (top) and dependence of the Lyapunov exponent *λ* (middle) and *F*(*f*_*max*,*min*_) + *K*(*u*(*f*_*max*,*min*_)) (bottom) on *K* for *a* = 6.02, 6.03, 6.04. From this result, we confirmed that the chaotic attractor (*λ* > 0) is divided in the regions for $K\gtrsim 0.045$ (*a* = 6.02), $K\gtrsim 0.062$ (*a* = 6.03), and $K\gtrsim 0.078$ (*a* = 6.04) due to the broken attractor merging condition: $F({f}_{{\rm{\max }}})+K(u({f}_{{\rm{\max }}}\mathrm{))} > \mathrm{0,}\,F({f}_{{\rm{\min }}})+K(u({f}_{{\rm{\min }}}\mathrm{))} < 0$. Within the region with chaos–chaos intermittency, *P*_*t*_ decreases with increasing *K*, that is, the frequency of chaos–chaos intermittency is reduced as the value of *K* increases.

### Controlling chaotic resonance

**Figure 4:**
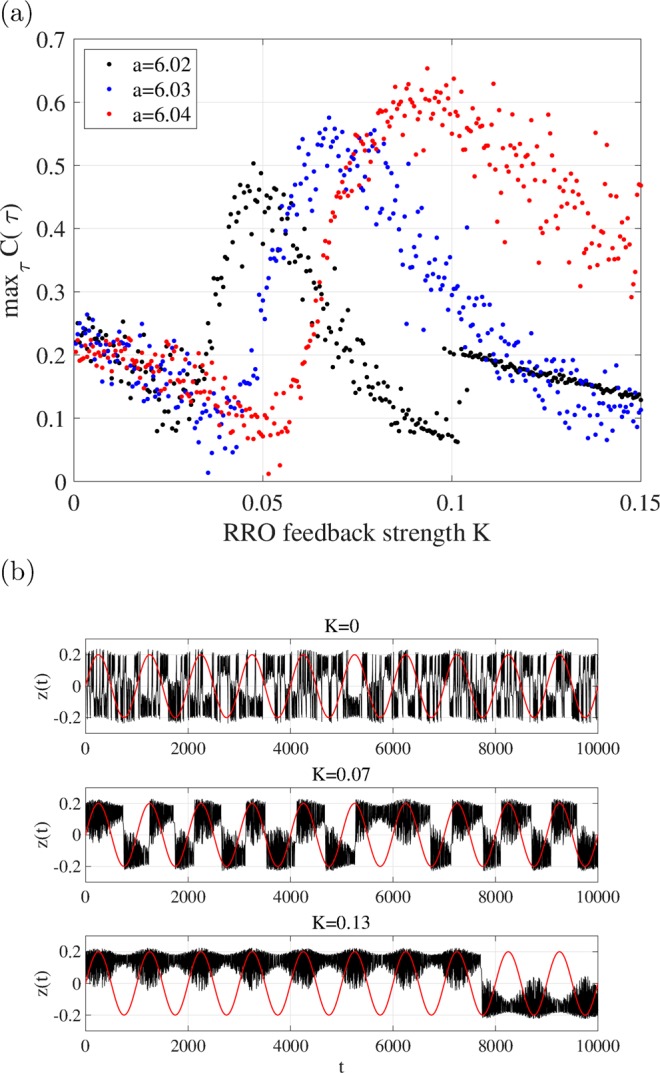
Dependence of signal response on RRO feedback strength *K*. (**a**) Dependence of correlation ${max}_{\tau }C(\tau )$ between input signal *S*(*t*) and binarised *z*(*t*) on *K*. (**b**) Time series of *z*(*t*) for *K* = 0 (top), *K* = 0.07 (middle), and *K* = 0.13 (bottom) when *a* = 6.03. The black and red lines indicate the time series of *z*(*t*) and *S*(*t*), respectively. The value of ${max}_{\tau }C(\tau )$ indicates a unimodal maximum peak around the attractor merging point given in Fig. 3.

In this section, the signal response of *z*(*t*) is evaluated with the input signal *A* = 0.02, *ω* = 10^−3^. Figure 4 shows the dependence of correlation max_*τ*_ *C*(*τ*) between input signal *S*(*t*) and the binarised *z*(*t*) on *K* and the typical time-series of *z*(*t*). The value of max_*τ*_ *C*(*τ*) indicates a unimodal maximum peak around the attractor merging point (*F*(*f*_*max*_) + *K*(*u*(*f*_*max*_)) = 0, *F*(*f*_*min*_) + *K*(*u*(*f*_*min*_)) = 0), that is, the chaotic resonance induced by the external feedback is confirmed.

### Signal response dependence on amplitude and frequency for input signal

**Figure 5:**
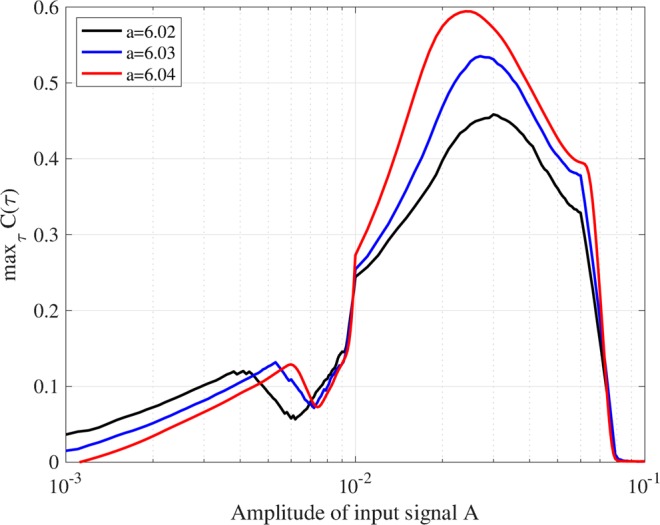
Dependence of signal response (correlation max_*τ*_ *C*(*τ*)) between input signal *S*(*t*) (*ω* = 10^−3^) and binarised *z*(*t*) on amplitude of input signal *A* for *a* = 6.02, 6.03, 6.04. *K* is set to values where max_*τ*_ *C*(*τ*) exhibits a peak in Fig. 4, that is, *K* = 0.05, 0.07, 0.09, for *a* = 6.02, 6.03, 6.04, respectively. The value for max_*τ*_ *C*(*τ*) exhibits a unimodal maximum peak against signal amplitude.

**Figure 6:**
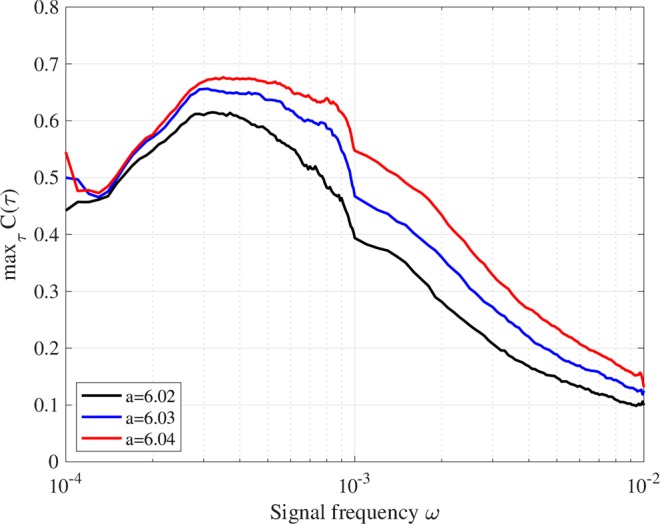
Dependence of signal response (correlation max_*τ*_ *C*(*τ*)) on signal frequency *ω* for *a* = 6.02, 6.03, 6.04. *K* is set to values where max_*τ*_ *C*(*τ*) exhibits a peak in Fig. 4, that is, *K* = 0.05, 0.07, 0.09, for *a* = 6.02, 6.03, 6.04, respectively. The value for max_*τ*_ *C*(*τ*) exhibits a peak at *ω* ≈ 3.0 × 10^−4^, that is, a resonance frequency in this neural system (*A* = 0.02).

It is known that chaotic resonance is induced in the regime of the weak amplitude of the input signal and exhibits signal response dependence on the frequency of the input signal^2,31,33,37^. In this section, we reviewed signal response dependence on these parameters of the input signal. Figure 5 shows the dependence of max_*τ*_ *C*(*τ*) between input signal *S*(*t*) (*ω* = 10^−3^) and binarised *z*(*t*) on the amplitude of input signal *A* when *a* = 6.02, 6.03, 6.04. Here, *K* is set to values where max_*τ*_ *C*(*τ*) exhibits the peak in Fig. 4, that is, *K* = 0.05, 0.07, 0.09 for *a* = 6.02, 6.03, 6.04, respectively. From this result, for all these values of *a*, max_*τ*_ *C*(*τ*) exhibits a unimodal maximum peak against signal amplitude. Figure 6 evaluates the dependence of max_*τ*_ *C*(*τ*) on signal frequency *ω* when *A* = 0.02. The result indicates that the value for max_*τ*_ *C*(*τ*) exhibits a peak at *ω* ≈ 3.0 × 10^−4^, that is, this frequency can be interpreted as a resonance frequency in this neural system.

### Signal response dependence on external noise

**Figure 7:**
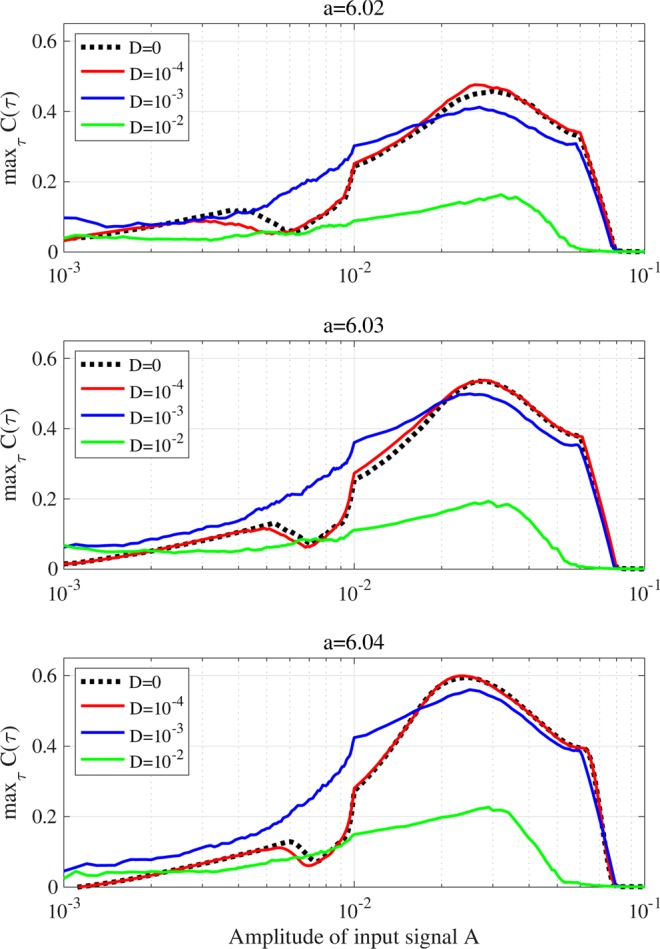
Dependence of signal response (correlation max_*τ*_ *C*(*τ*)) on amplitude of input signal *A* in the presence of Gaussian white noise (noise strength: *D* = 10^−4^, 10^−3^, 10^−2^) for *a* = 6.02, 6.03, 6.04. The black dotted lines indicate values for max_*τ*_ *C*(*τ*) in the noise-free condition corresponding to Fig. 5. The decreasing trend of max_*τ*_ *C*(*τ*) with increasing noise strength *D* at *A* ≈ 0.03 and the noise enhancement of signal response (${10}^{-3}\le A\lesssim {10}^{-2}$, *D* = 10^−3^) are confirmed.

Background stochastic noise is assumed to exist in an actual neural system. Therefore, its influence on chaotic resonance needs to be evaluated. Figure 7 shows the dependence of max_*τ*_ *C*(*τ*) on signal amplitude *A* under Gaussian white noise *Dξ* (*t*). The decreasing trend of max_*τ*_ *C*(*τ*) with increasing noise strength *D* at *A* ≈ 0.03 is confirmed. In the weak signal strength region ${10}^{-3}\le A\lesssim {10}^{-2}$, the values for max_*τ*_ *C*(*τ*) at *D* = 10^−3^ increase in comparison with the noise-free condition.

## Discussion and Conclusion

In this study, we applied the RRO method to a neural system consisting of excitatory and inhibitory neurons and controlled attractor merging for the orbit of effective neural potential in excitatory and inhibitory neurons. Then, by adjusting the feedback of the RRO method to an appropriate strength, we maximised the response to a weak sinusoidal signal. This response possesses the characteristics of a resonance frequency. Furthermore, the presence of noise exhibits effects for both diminishing and enhancing the signal response in chaotic resonance.

In conventional chaos control methods, such as the Ott–Grebogi–Yorke^38^, delayed feedback^39,40^, and *H*_∞_ control^41^ methods, chaotic states should be eliminated by stabilising the equilibrium and transiting to a periodic state through external perturbations, under the assumption that chaotic states degrade system performance. On the contrary, in the RRO method, the chaotic attractor is merged with chaos–chaos intermittency by adjusting feedback strength, maintaining the chaotic state. Consequently, at a feedback strength close to that required for attractor merging, the response to weak input signals is maximised; instead of degrading the signal response, chaotic resonance is induced. This finding is consistent with our previous work dealing with controlling attractor merging and chaotic resonance in a simple cubic map model^37,42^.

Zambrano *et al*. devised another similar solution for controlling chaotic resonance by developing a novel technique for inducing chaotic resonance using the Ott–Grebogi–Yorke method^29^. In comparison with our study, the chaotic resonance they focused on is applied to external additive chaotic signals, and chaotic resonance is induced by stabilising the external additive chaotic signal to the periodic signal, with the period corresponding to the input signal. While we dealt with chaotic resonance using internal chaotic dynamics, the resonance itself is induced by controlling the chaotic attractor merging instead of stabilising the chaotic state.

Regarding the characteristics of the signal response in the chaotic resonance, chaotic resonance is induced in the weak signal amplitude region and possesses resonance frequency. These fundamental characteristics are also consistent with the findings of our previous study^7,33^. Furthermore, the influence of external noise should be discussed. In the relatively strong signal amplitude region (*A* ≈ 0.03), the signal response of chaotic resonance is degraded by external noise because a chaotic orbit with instability is easily disturbed by external perturbation (see Fig. 7). In contrast, in the relatively weak signal strength region (${10}^{-3}\le A\lesssim {10}^{-2}$), the signal response is enhanced at intermediate noise strength (*D* = 10^−3^). A plausible reason for this enhancement is that stochastic noise can enhance the signal response at appropriate noise strengths in chaotic systems, that is, stochastic resonance can arise in chaotic systems^2^. Therefore, the enhancement of signal response by external noise, observed in Fig. 7, may be interpreted as the effect of stochastic resonance in a chaotic system.

Some limitations of this study must be considered. The neural system used in this study is the simplest model with a discrete map structure for chaos–chaos intermittency. Therefore, to apply the RRO method to an actual neural system, such as sensory and brain neural systems, chaotic resonance induced by the RRO method must be evaluated in more complex and continuous neural models. Further, the design of an RRO feedback controller for actual neural systems is vital for this application. Additionally, controlling chaotic resonance must be considered not only at the level of neurons or small assemblies, but also in large neural networks.

In conclusion, this study shows that chaotic resonance can be induced by an external feedback signal based on the RRO method in neural systems. Although several limitations remain, the outcome of this study might facilitate the development of devices for enhancing signal response using the mechanism of chaotic resonance in actual neural systems whose internal parameters cannot be adjusted externally.
